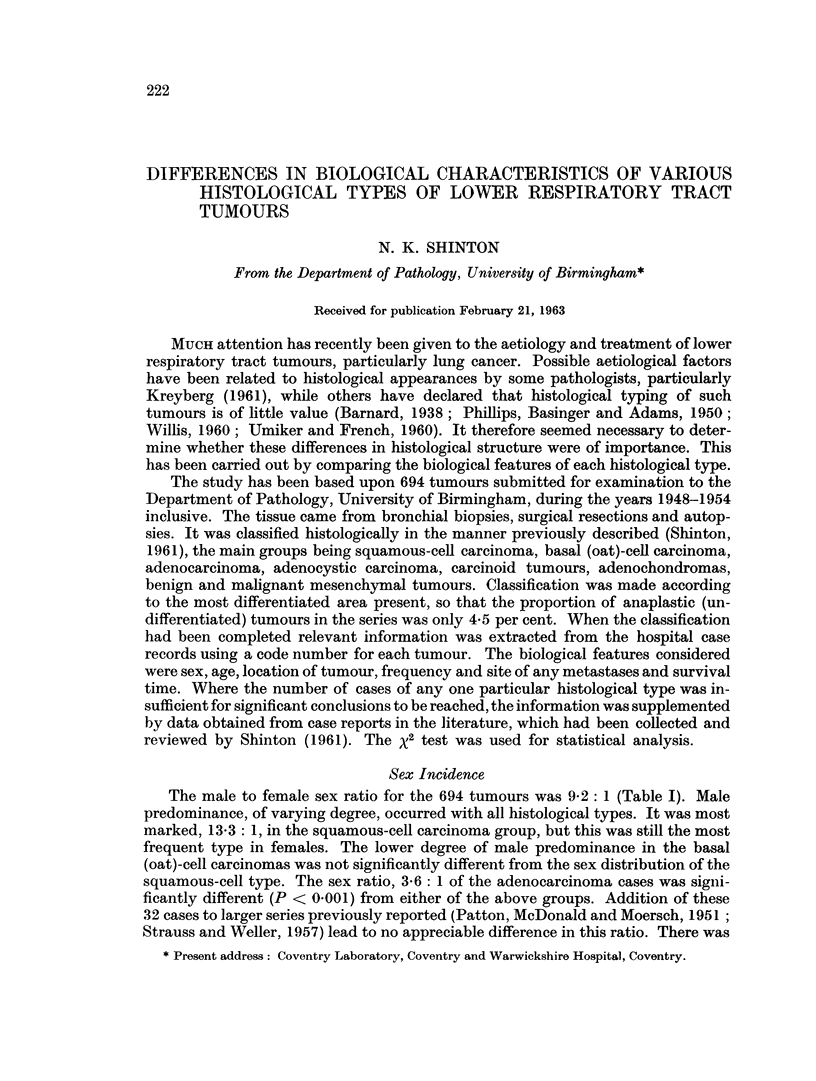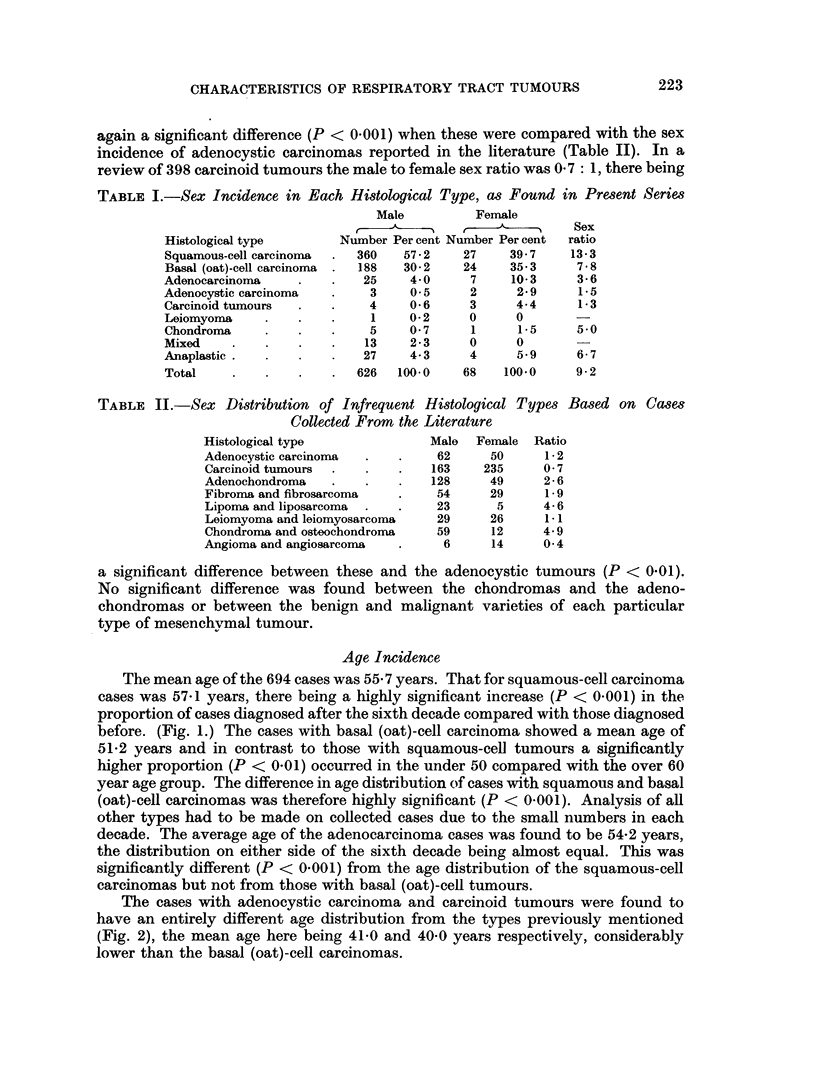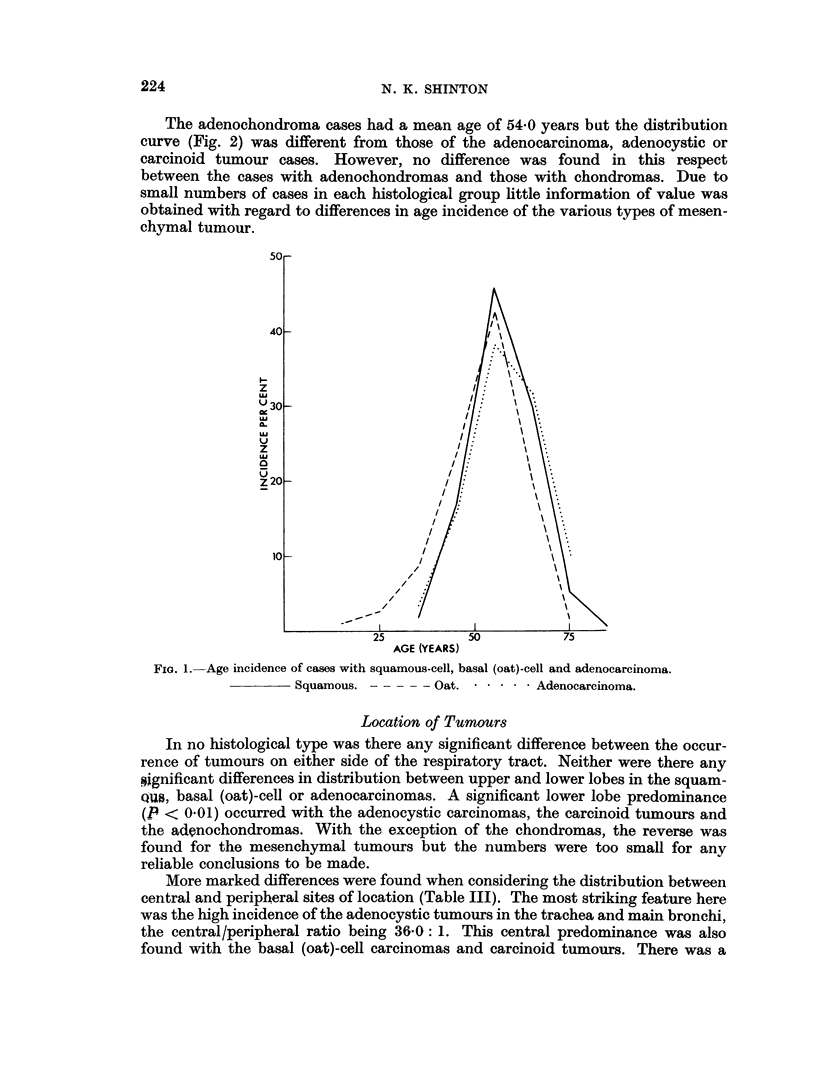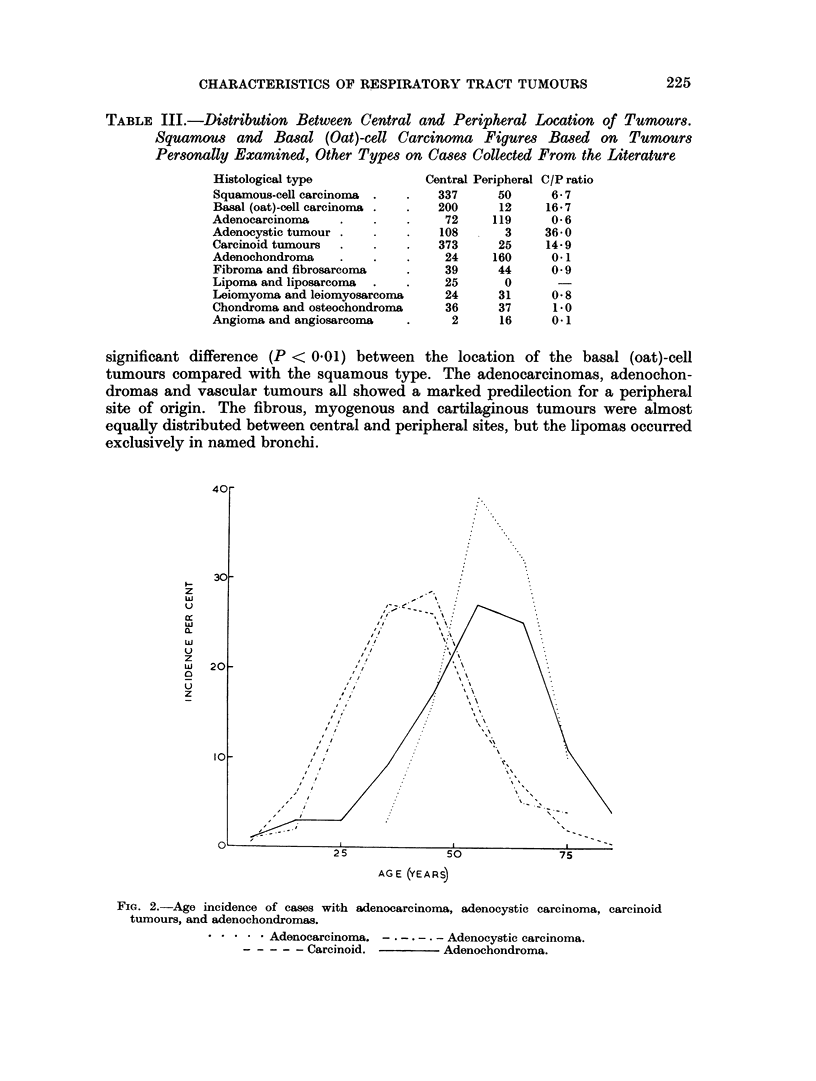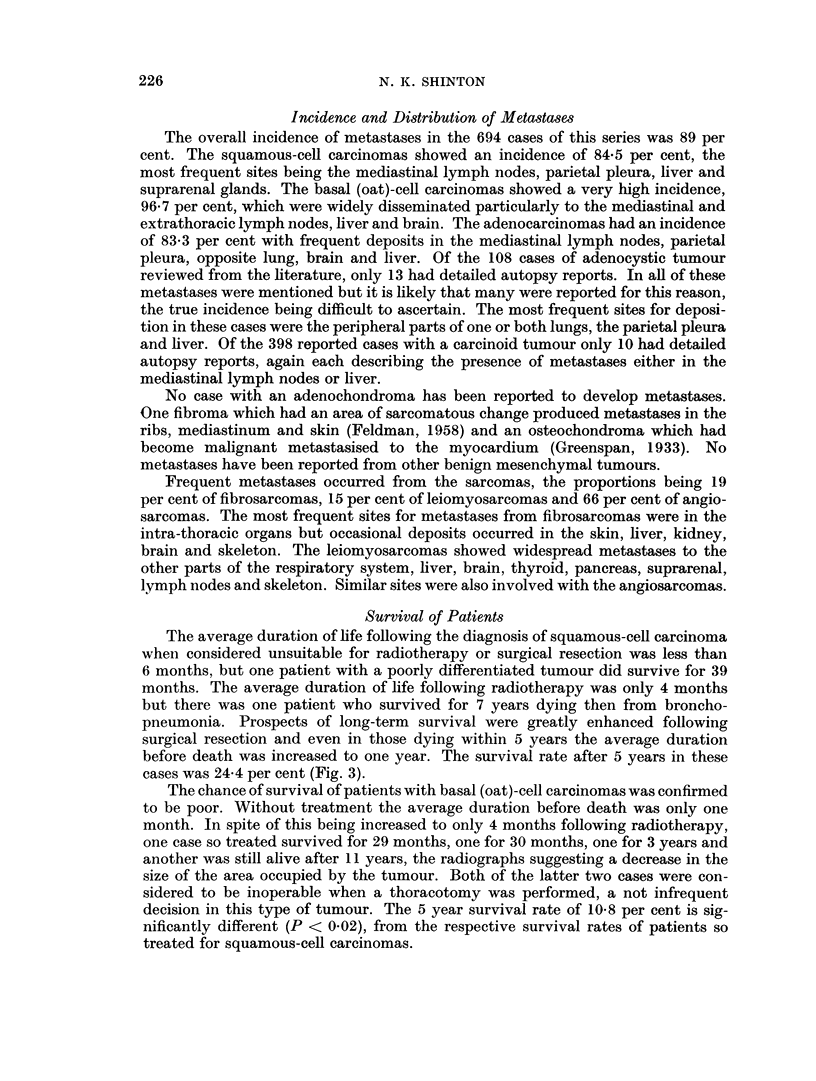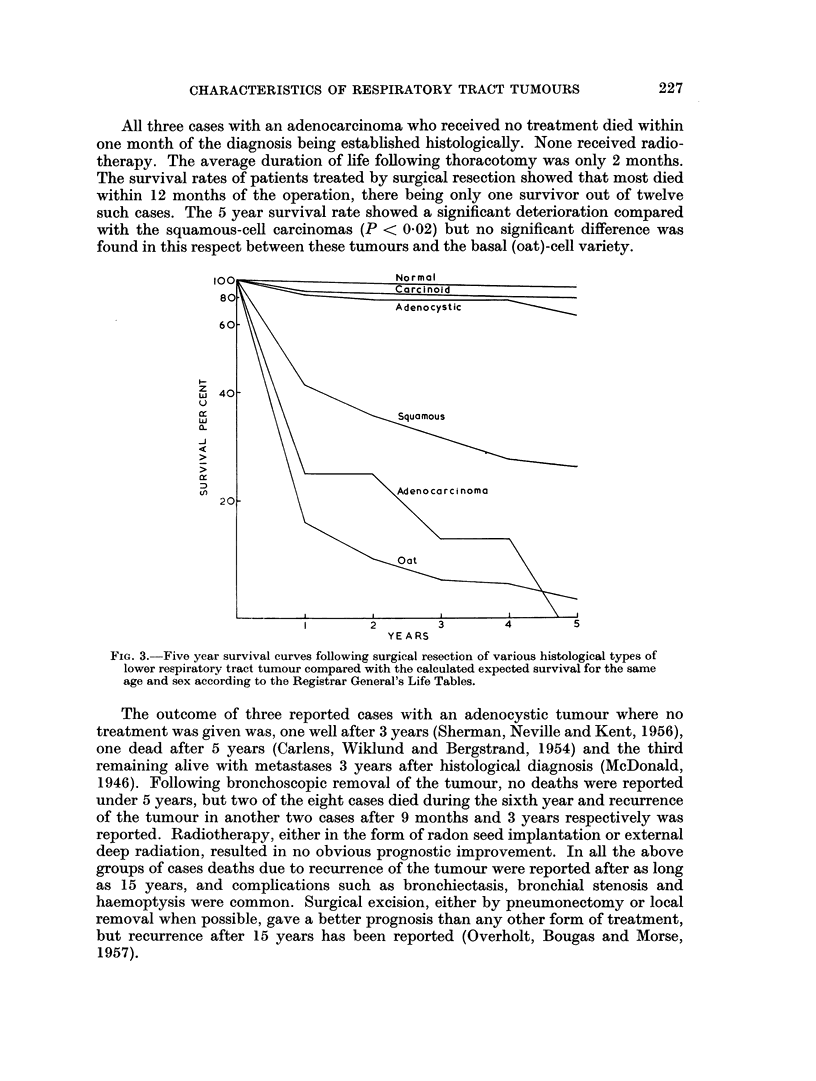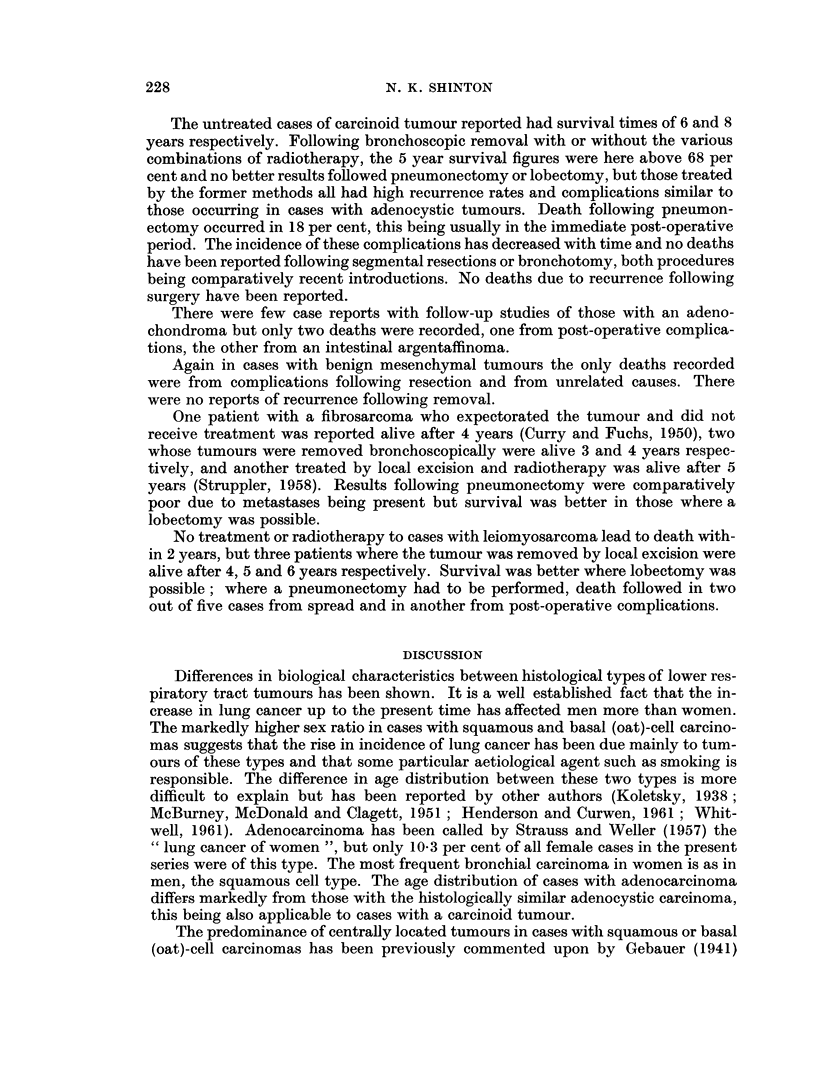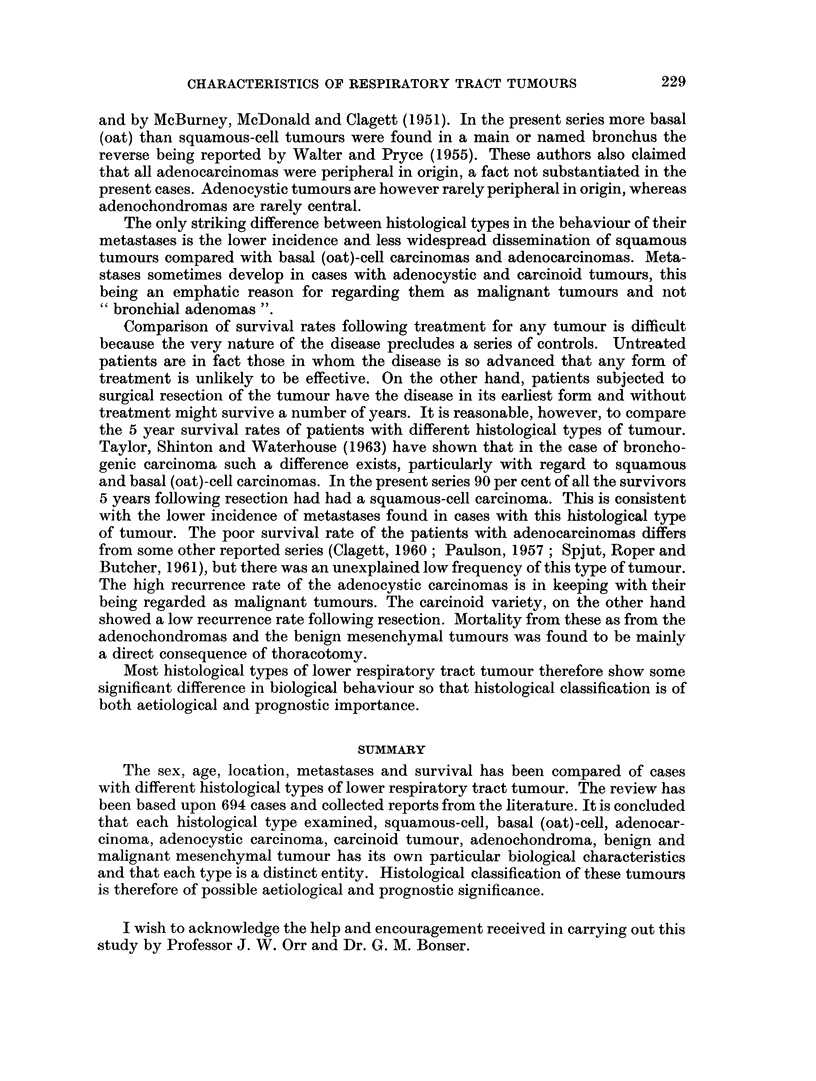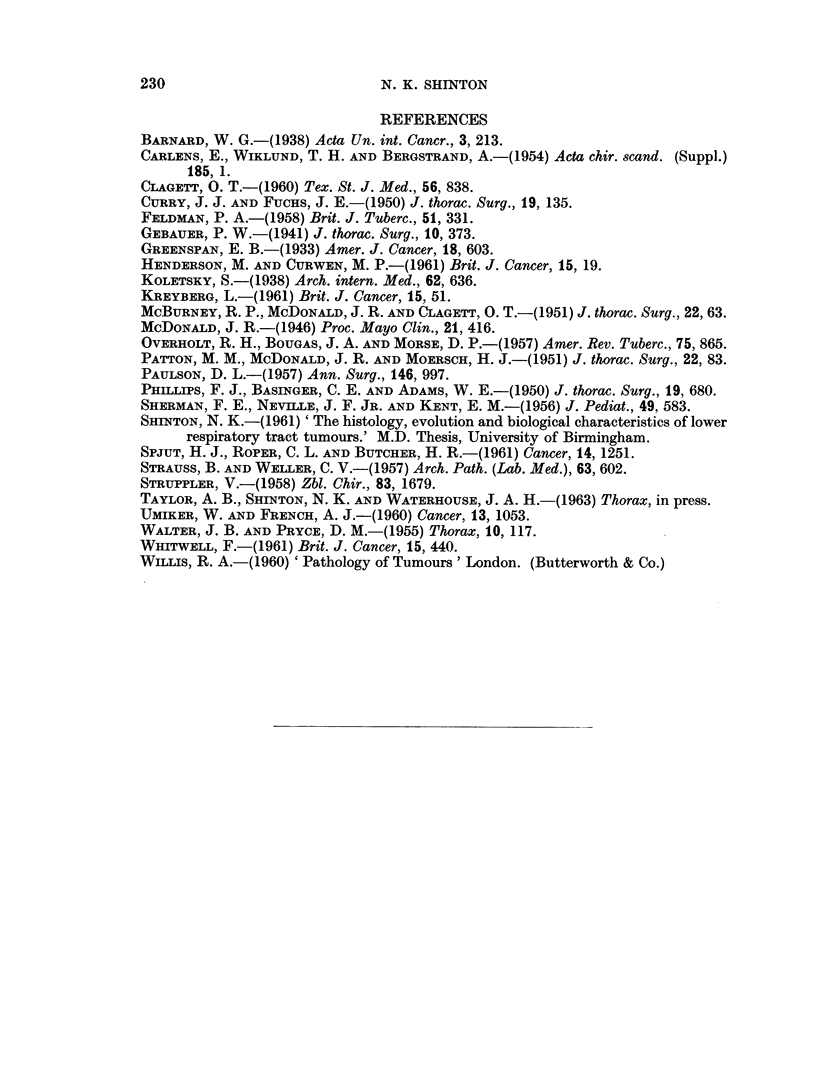# Differences in Biological Characteristics of Various Histological Types of Lower Respiratory Tract Tumours

**DOI:** 10.1038/bjc.1963.31

**Published:** 1963-06

**Authors:** N. K. Shinton


### DIFFERENCES IN BIOLOGICAL CHARACTERISTICS OF VARIOUS HISTOLOGICAL TYPES OF LOWER RESPIRATORY TRACT TUMOURS

N. K. SHINTON

*From the Department of Pathology, University of Birmingham**

Received for publication February 21, 1963

MUCH attention has recently been given to the aetiology and treatment of lower respiratory tract tumours, particularly lung cancer. Possible aetiological factors have been related to histological appearances by some pathologists, particularly Kreyberg (1961), while others have declared that histological typing of such tumours is of little value (Barnard, 1938 ; Phillips, Basinger and Adams, 1950 ; Willis, 1960 ; Umiker and French, 1960). It therefore seemed necessary to determine whether these differences in histological structure were of importance. This has been carried out by comparing the biological features of each histological type.

The study has been based upon 694 tumours submitted for examination to the Department of Pathology, University of Birmingham, during the years 1948–1954 inclusive. The tissue came from bronchial biopsies, surgical resections and autopsies. It was classified histologically in the manner previously described (Shinton, 1961), the main groups being squamous-cell carcinoma, basal (oat)-cell carcinoma, adenocarcinoma, adenocystic carcinoma, carcinoid tumours, adenochondromas, benign and malignant mesenchymal tumours. Classification was made according to the most differentiated area present, so that the proportion of anaplastic (undifferentiated) tumours in the series was only 4·5 per cent. When the classification had been completed relevant information was extracted from the hospital case records using a code number for each tumour. The biological features considered were sex, age, location of tumour, frequency and site of any metastases and survival time. Where the number of cases of any one particular histological type was insufficient for significant conclusions to be reached, the information was supplemented by data obtained from case reports in the literature, which had been collected and reviewed by Shinton (1961). The $\chi^2$ test was used for statistical analysis.

### *Sex Incidence*

The male to female sex ratio for the 694 tumours was 9·2 : 1 (Table I). Male predominance, of varying degree, occurred with all histological types. It was most marked, 13·3 : 1, in the squamous-cell carcinoma group, but this was still the most frequent type in females. The lower degree of male predominance in the basal (oat)-cell carcinomas was not significantly different from the sex distribution of the squamous-cell type. The sex ratio, 3·6 : 1 of the adenocarcinoma cases was significantly different ($P < 0{\cdot}001$) from either of the above groups. Addition of these 32 cases to larger series previously reported (Patton, McDonald and Moersch, 1951 ; Strauss and Weller, 1957) lead to no appreciable difference in this ratio. There was

* Present address : Coventry Laboratory, Coventry and Warwickshire Hospital, Coventry.

again a significant difference ($P < 0{\cdot}001$) when these were compared with the sex incidence of adenocystic carcinomas reported in the literature (Table II). In a review of 398 carcinoid tumours the male to female sex ratio was 0·7 : 1, there being

TABLE I.—*Sex Incidence in Each Histological Type, as Found in Present Series*

| Histological type | Male Number | Male Per cent | Female Number | Female Per cent | Sex ratio |
|---|---|---|---|---|---|
| Squamous-cell carcinoma | 360 | 57·2 | 27 | 39·7 | 13·3 |
| Basal (oat)-cell carcinoma | 188 | 30·2 | 24 | 35·3 | 7·8 |
| Adenocarcinoma | 25 | 4·0 | 7 | 10·3 | 3·6 |
| Adenocystic carcinoma | 3 | 0·5 | 2 | 2·9 | 1·5 |
| Carcinoid tumours | 4 | 0·6 | 3 | 4·4 | 1·3 |
| Leiomyoma | 1 | 0·2 | 0 | 0 | — |
| Chondroma | 5 | 0·7 | 1 | 1·5 | 5·0 |
| Mixed | 13 | 2·3 | 0 | 0 | — |
| Anaplastic | 27 | 4·3 | 4 | 5·9 | 6·7 |
| Total | 626 | 100·0 | 68 | 100·0 | 9·2 |

TABLE II.—*Sex Distribution of Infrequent Histological Types Based on Cases Collected From the Literature*

| Histological type | Male | Female | Ratio |
|---|---|---|---|
| Adenocystic carcinoma | 62 | 50 | 1·2 |
| Carcinoid tumours | 163 | 235 | 0·7 |
| Adenochondroma | 128 | 49 | 2·6 |
| Fibroma and fibrosarcoma | 54 | 29 | 1·9 |
| Lipoma and liposarcoma | 23 | 5 | 4·6 |
| Leiomyoma and leiomyosarcoma | 29 | 26 | 1·1 |
| Chondroma and osteochondroma | 59 | 12 | 4·9 |
| Angioma and angiosarcoma | 6 | 14 | 0·4 |

a significant difference between these and the adenocystic tumours ($P < 0{\cdot}01$). No significant difference was found between the chondromas and the adenochondromas or between the benign and malignant varieties of each particular type of mesenchymal tumour.

### *Age Incidence*

The mean age of the 694 cases was 55·7 years. That for squamous-cell carcinoma cases was 57·1 years, there being a highly significant increase ($P < 0{\cdot}001$) in the proportion of cases diagnosed after the sixth decade compared with those diagnosed before. (Fig. 1.) The cases with basal (oat)-cell carcinoma showed a mean age of 51·2 years and in contrast to those with squamous-cell tumours a significantly higher proportion ($P < 0{\cdot}01$) occurred in the under 50 compared with the over 60 year age group. The difference in age distribution of cases with squamous and basal (oat)-cell carcinomas was therefore highly significant ($P < 0{\cdot}001$). Analysis of all other types had to be made on collected cases due to the small numbers in each decade. The average age of the adenocarcinoma cases was found to be 54·2 years, the distribution on either side of the sixth decade being almost equal. This was significantly different ($P < 0{\cdot}001$) from the age distribution of the squamous-cell carcinomas but not from those with basal (oat)-cell tumours.

The cases with adenocystic carcinoma and carcinoid tumours were found to have an entirely different age distribution from the types previously mentioned (Fig. 2), the mean age here being 41·0 and 40·0 years respectively, considerably lower than the basal (oat)-cell carcinomas.

The adenochondroma cases had a mean age of 54·0 years but the distribution curve (Fig. 2) was different from those of the adenocarcinoma, adenocystic or carcinoid tumour cases. However, no difference was found in this respect between the cases with adenochondromas and those with chondromas. Due to small numbers of cases in each histological group little information of value was obtained with regard to differences in age incidence of the various types of mesenchymal tumour.

FIG. 1.—Age incidence of cases with squamous-cell, basal (oat)-cell and adenocarcinoma. ———— Squamous. – – – – – Oat. · · · · · Adenocarcinoma.

*Location of Tumours*

In no histological type was there any significant difference between the occurrence of tumours on either side of the respiratory tract. Neither were there any significant differences in distribution between upper and lower lobes in the squamous, basal (oat)-cell or adenocarcinomas. A significant lower lobe predominance ($P < 0{\cdot}01$) occurred with the adenocystic carcinomas, the carcinoid tumours and the adenochondromas. With the exception of the chondromas, the reverse was found for the mesenchymal tumours but the numbers were too small for any reliable conclusions to be made.

More marked differences were found when considering the distribution between central and peripheral sites of location (Table III). The most striking feature here was the high incidence of the adenocystic tumours in the trachea and main bronchi, the central/peripheral ratio being 36·0 : 1. This central predominance was also found with the basal (oat)-cell carcinomas and carcinoid tumours. There was a

TABLE III.—*Distribution Between Central and Peripheral Location of Tumours. Squamous and Basal (Oat)-cell Carcinoma Figures Based on Tumours Personally Examined, Other Types on Cases Collected From the Literature*

| Histological type | Central | Peripheral | C/P ratio |
|---|---|---|---|
| Squamous-cell carcinoma . . | 337 | 50 | 6·7 |
| Basal (oat)-cell carcinoma . . | 200 | 12 | 16·7 |
| Adenocarcinoma . . . | 72 | 119 | 0·6 |
| Adenocystic tumour . . . | 108 | 3 | 36·0 |
| Carcinoid tumours . . . | 373 | 25 | 14·9 |
| Adenochondroma . . . | 24 | 160 | 0·1 |
| Fibroma and fibrosarcoma . | 39 | 44 | 0·9 |
| Lipoma and liposarcoma . . | 25 | 0 | — |
| Leiomyoma and leiomyosarcoma | 24 | 31 | 0·8 |
| Chondroma and osteochondroma | 36 | 37 | 1·0 |
| Angioma and angiosarcoma . | 2 | 16 | 0·1 |

significant difference ($P < 0{\cdot}01$) between the location of the basal (oat)-cell tumours compared with the squamous type. The adenocarcinomas, adenochondromas and vascular tumours all showed a marked predilection for a peripheral site of origin. The fibrous, myogenous and cartilaginous tumours were almost equally distributed between central and peripheral sites, but the lipomas occurred exclusively in named bronchi.

FIG. 2.—Age incidence of cases with adenocarcinoma, adenocystic carcinoma, carcinoid tumours, and adenochondromas.

· · · · · Adenocarcinoma. — · — · — · — Adenocystic carcinoma.
— — — — — Carcinoid. ———— Adenochondroma.

### *Incidence and Distribution of Metastases*

The overall incidence of metastases in the 694 cases of this series was 89 per cent. The squamous-cell carcinomas showed an incidence of 84·5 per cent, the most frequent sites being the mediastinal lymph nodes, parietal pleura, liver and suprarenal glands. The basal (oat)-cell carcinomas showed a very high incidence, 96·7 per cent, which were widely disseminated particularly to the mediastinal and extrathoracic lymph nodes, liver and brain. The adenocarcinomas had an incidence of 83·3 per cent with frequent deposits in the mediastinal lymph nodes, parietal pleura, opposite lung, brain and liver. Of the 108 cases of adenocystic tumour reviewed from the literature, only 13 had detailed autopsy reports. In all of these metastases were mentioned but it is likely that many were reported for this reason, the true incidence being difficult to ascertain. The most frequent sites for deposition in these cases were the peripheral parts of one or both lungs, the parietal pleura and liver. Of the 398 reported cases with a carcinoid tumour only 10 had detailed autopsy reports, again each describing the presence of metastases either in the mediastinal lymph nodes or liver.

No case with an adenochondroma has been reported to develop metastases. One fibroma which had an area of sarcomatous change produced metastases in the ribs, mediastinum and skin (Feldman, 1958) and an osteochondroma which had become malignant metastasised to the myocardium (Greenspan, 1933). No metastases have been reported from other benign mesenchymal tumours.

Frequent metastases occurred from the sarcomas, the proportions being 19 per cent of fibrosarcomas, 15 per cent of leiomyosarcomas and 66 per cent of angiosarcomas. The most frequent sites for metastases from fibrosarcomas were in the intra-thoracic organs but occasional deposits occurred in the skin, liver, kidney, brain and skeleton. The leiomyosarcomas showed widespread metastases to the other parts of the respiratory system, liver, brain, thyroid, pancreas, suprarenal, lymph nodes and skeleton. Similar sites were also involved with the angiosarcomas.

### *Survival of Patients*

The average duration of life following the diagnosis of squamous-cell carcinoma when considered unsuitable for radiotherapy or surgical resection was less than 6 months, but one patient with a poorly differentiated tumour did survive for 39 months. The average duration of life following radiotherapy was only 4 months but there was one patient who survived for 7 years dying then from bronchopneumonia. Prospects of long-term survival were greatly enhanced following surgical resection and even in those dying within 5 years the average duration before death was increased to one year. The survival rate after 5 years in these cases was 24·4 per cent (Fig. 3).

The chance of survival of patients with basal (oat)-cell carcinomas was confirmed to be poor. Without treatment the average duration before death was only one month. In spite of this being increased to only 4 months following radiotherapy, one case so treated survived for 29 months, one for 30 months, one for 3 years and another was still alive after 11 years, the radiographs suggesting a decrease in the size of the area occupied by the tumour. Both of the latter two cases were considered to be inoperable when a thoracotomy was performed, a not infrequent decision in this type of tumour. The 5 year survival rate of 10·8 per cent is significantly different ($P < 0{\cdot}02$), from the respective survival rates of patients so treated for squamous-cell carcinomas.

All three cases with an adenocarcinoma who received no treatment died within one month of the diagnosis being established histologically. None received radiotherapy. The average duration of life following thoracotomy was only 2 months. The survival rates of patients treated by surgical resection showed that most died within 12 months of the operation, there being only one survivor out of twelve such cases. The 5 year survival rate showed a significant deterioration compared with the squamous-cell carcinomas ($P < 0{\cdot}02$) but no significant difference was found in this respect between these tumours and the basal (oat)-cell variety.

FIG. 3.—Five year survival curves following surgical resection of various histological types of lower respiratory tract tumour compared with the calculated expected survival for the same age and sex according to the Registrar General's Life Tables.

The outcome of three reported cases with an adenocystic tumour where no treatment was given was, one well after 3 years (Sherman, Neville and Kent, 1956), one dead after 5 years (Carlens, Wiklund and Bergstrand, 1954) and the third remaining alive with metastases 3 years after histological diagnosis (McDonald, 1946). Following bronchoscopic removal of the tumour, no deaths were reported under 5 years, but two of the eight cases died during the sixth year and recurrence of the tumour in another two cases after 9 months and 3 years respectively was reported. Radiotherapy, either in the form of radon seed implantation or external deep radiation, resulted in no obvious prognostic improvement. In all the above groups of cases deaths due to recurrence of the tumour were reported after as long as 15 years, and complications such as bronchiectasis, bronchial stenosis and haemoptysis were common. Surgical excision, either by pneumonectomy or local removal when possible, gave a better prognosis than any other form of treatment, but recurrence after 15 years has been reported (Overholt, Bougas and Morse, 1957).

The untreated cases of carcinoid tumour reported had survival times of 6 and 8 years respectively. Following bronchoscopic removal with or without the various combinations of radiotherapy, the 5 year survival figures were here above 68 per cent and no better results followed pneumonectomy or lobectomy, but those treated by the former methods all had high recurrence rates and complications similar to those occurring in cases with adenocystic tumours. Death following pneumonectomy occurred in 18 per cent, this being usually in the immediate post-operative period. The incidence of these complications has decreased with time and no deaths have been reported following segmental resections or bronchotomy, both procedures being comparatively recent introductions. No deaths due to recurrence following surgery have been reported.

There were few case reports with follow-up studies of those with an adenochondroma but only two deaths were recorded, one from post-operative complications, the other from an intestinal argentaffinoma.

Again in cases with benign mesenchymal tumours the only deaths recorded were from complications following resection and from unrelated causes. There were no reports of recurrence following removal.

One patient with a fibrosarcoma who expectorated the tumour and did not receive treatment was reported alive after 4 years (Curry and Fuchs, 1950), two whose tumours were removed bronchoscopically were alive 3 and 4 years respectively, and another treated by local excision and radiotherapy was alive after 5 years (Struppler, 1958). Results following pneumonectomy were comparatively poor due to metastases being present but survival was better in those where a lobectomy was possible.

No treatment or radiotherapy to cases with leiomyosarcoma lead to death within 2 years, but three patients where the tumour was removed by local excision were alive after 4, 5 and 6 years respectively. Survival was better where lobectomy was possible ; where a pneumonectomy had to be performed, death followed in two out of five cases from spread and in another from post-operative complications.

### DISCUSSION

Differences in biological characteristics between histological types of lower respiratory tract tumours has been shown. It is a well established fact that the increase in lung cancer up to the present time has affected men more than women. The markedly higher sex ratio in cases with squamous and basal (oat)-cell carcinomas suggests that the rise in incidence of lung cancer has been due mainly to tumours of these types and that some particular aetiological agent such as smoking is responsible. The difference in age distribution between these two types is more difficult to explain but has been reported by other authors (Koletsky, 1938 ; McBurney, McDonald and Clagett, 1951 ; Henderson and Curwen, 1961 ; Whitwell, 1961). Adenocarcinoma has been called by Strauss and Weller (1957) the " lung cancer of women ", but only 10·3 per cent of all female cases in the present series were of this type. The most frequent bronchial carcinoma in women is as in men, the squamous cell type. The age distribution of cases with adenocarcinoma differs markedly from those with the histologically similar adenocystic carcinoma, this being also applicable to cases with a carcinoid tumour.

The predominance of centrally located tumours in cases with squamous or basal (oat)-cell carcinomas has been previously commented upon by Gebauer (1941)

and by McBurney, McDonald and Clagett (1951). In the present series more basal (oat) than squamous-cell tumours were found in a main or named bronchus the reverse being reported by Walter and Pryce (1955). These authors also claimed that all adenocarcinomas were peripheral in origin, a fact not substantiated in the present cases. Adenocystic tumours are however rarely peripheral in origin, whereas adenochondromas are rarely central.

The only striking difference between histological types in the behaviour of their metastases is the lower incidence and less widespread dissemination of squamous tumours compared with basal (oat)-cell carcinomas and adenocarcinomas. Metastases sometimes develop in cases with adenocystic and carcinoid tumours, this being an emphatic reason for regarding them as malignant tumours and not " bronchial adenomas ".

Comparison of survival rates following treatment for any tumour is difficult because the very nature of the disease precludes a series of controls. Untreated patients are in fact those in whom the disease is so advanced that any form of treatment is unlikely to be effective. On the other hand, patients subjected to surgical resection of the tumour have the disease in its earliest form and without treatment might survive a number of years. It is reasonable, however, to compare the 5 year survival rates of patients with different histological types of tumour. Taylor, Shinton and Waterhouse (1963) have shown that in the case of bronchogenic carcinoma such a difference exists, particularly with regard to squamous and basal (oat)-cell carcinomas. In the present series 90 per cent of all the survivors 5 years following resection had had a squamous-cell carcinoma. This is consistent with the lower incidence of metastases found in cases with this histological type of tumour. The poor survival rate of the patients with adenocarcinomas differs from some other reported series (Clagett, 1960 ; Paulson, 1957 ; Spjut, Roper and Butcher, 1961), but there was an unexplained low frequency of this type of tumour. The high recurrence rate of the adenocystic carcinomas is in keeping with their being regarded as malignant tumours. The carcinoid variety, on the other hand showed a low recurrence rate following resection. Mortality from these as from the adenochondromas and the benign mesenchymal tumours was found to be mainly a direct consequence of thoracotomy.

Most histological types of lower respiratory tract tumour therefore show some significant difference in biological behaviour so that histological classification is of both aetiological and prognostic importance.

### SUMMARY

The sex, age, location, metastases and survival has been compared of cases with different histological types of lower respiratory tract tumour. The review has been based upon 694 cases and collected reports from the literature. It is concluded that each histological type examined, squamous-cell, basal (oat)-cell, adenocarcinoma, adenocystic carcinoma, carcinoid tumour, adenochondroma, benign and malignant mesenchymal tumour has its own particular biological characteristics and that each type is a distinct entity. Histological classification of these tumours is therefore of possible aetiological and prognostic significance.

I wish to acknowledge the help and encouragement received in carrying out this study by Professor J. W. Orr and Dr. G. M. Bonser.

### REFERENCES

BARNARD, W. G.—(1938) *Acta Un. int. Cancr.*, **3**, 213.
CARLENS, E., WIKLUND, T. H. AND BERGSTRAND, A.—(1954) *Acta chir. scand.* (Suppl.) **185**, 1.
CLAGETT, O. T.—(1960) *Tex. St. J. Med.*, **56**, 838.
CURRY, J. J. AND FUCHS, J. E.—(1950) *J. thorac. Surg.*, **19**, 135.
FELDMAN, P. A.—(1958) *Brit. J. Tuberc.*, **51**, 331.
GEBAUER, P. W.—(1941) *J. thorac. Surg.*, **10**, 373.
GREENSPAN, E. B.—(1933) *Amer. J. Cancer*, **18**, 603.
HENDERSON, M. AND CURWEN, M. P.—(1961) *Brit. J. Cancer*, **15**, 19.
KOLETSKY, S.—(1938) *Arch. intern. Med.*, **62**, 636.
KREYBERG, L.—(1961) *Brit. J. Cancer*, **15**, 51.
MCBURNEY, R. P., MCDONALD, J. R. AND CLAGETT, O. T.—(1951) *J. thorac. Surg.*, **22**, 63.
MCDONALD, J. R.—(1946) *Proc. Mayo Clin.*, **21**, 416.
OVERHOLT, R. H., BOUGAS, J. A. AND MORSE, D. P.—(1957) *Amer. Rev. Tuberc.*, **75**, 865.
PATTON, M. M., MCDONALD, J. R. AND MOERSCH, H. J.—(1951) *J. thorac. Surg.*, **22**, 83.
PAULSON, D. L.—(1957) *Ann. Surg.*, **146**, 997.
PHILLIPS, F. J., BASINGER, C. E. AND ADAMS, W. E.—(1950) *J. thorac. Surg.*, **19**, 680.
SHERMAN, F. E., NEVILLE, J. F. JR. AND KENT, E. M.—(1956) *J. Pediat.*, **49**, 583.
SHINTON, N. K.—(1961) 'The histology, evolution and biological characteristics of lower respiratory tract tumours.' M.D. Thesis, University of Birmingham.
SPJUT, H. J., ROPER, C. L. AND BUTCHER, H. R.—(1961) *Cancer*, **14**, 1251.
STRAUSS, B. AND WELLER, C. V.—(1957) *Arch. Path.* (*Lab. Med.*), **63**, 602.
STRUPPLER, V.—(1958) *Zbl. Chir.*, **83**, 1679.
TAYLOR, A. B., SHINTON, N. K. AND WATERHOUSE, J. A. H.—(1963) *Thorax*, in press.
UMIKER, W. AND FRENCH, A. J.—(1960) *Cancer*, **13**, 1053.
WALTER, J. B. AND PRYCE, D. M.—(1955) *Thorax*, **10**, 117.
WHITWELL, F.—(1961) *Brit. J. Cancer*, **15**, 440.
WILLIS, R. A.—(1960) 'Pathology of Tumours' London. (Butterworth & Co.)